# Identification of gravid mosquitoes from changes in spectral and polarimetric backscatter cross sections

**DOI:** 10.1002/jbio.201900123

**Published:** 2019-07-15

**Authors:** Adrien P. Genoud, Yunpeng Gao, Gregory M. Williams, Benjamin P. Thomas

**Affiliations:** ^1^ Department of Physics, New Jersey Institute of Technology Newark New Jersey; ^2^ Center for Vector Biology Rutgers University New Brunswick New Jersey

**Keywords:** classification, entomology, gravid, lidar, mosquito, remote sensing

## Abstract

Improving the survey of mosquito populations is of the utmost importance to further enhance mitigation techniques that protect human populations from mosquito‐borne diseases. While mosquito populations are generally studied using physical traps, stand‐off optical sensors allow to study insect ecosystems with potentially better spatial and temporal resolution. This can be greatly beneficial to eco‐epidemiological models and various mosquito control programs. In this contribution, we demonstrate that the gravidity of female mosquitoes can be identified from changes in their spectral and polarimetric backscatter cross sections. Among other predictive variables, the wing beat frequency and the depolarization ratio of the mosquito body allows for the identification of gravid females with a precision and recall of 86% and 87%, respectively. Since female mosquitoes need a blood meal to become gravid, statistics on gravidity is of prime importance as only females that have been gravid might carry infectious diseases. In addition, it allows to detect possible breeding habitat, predict a potential increase in the mosquito population and provide a better overall understanding of the ecosystem dynamics. As a result, targeted and localized mitigation techniques can be used, reducing the cost and improving the efficiency of mosquito population control.
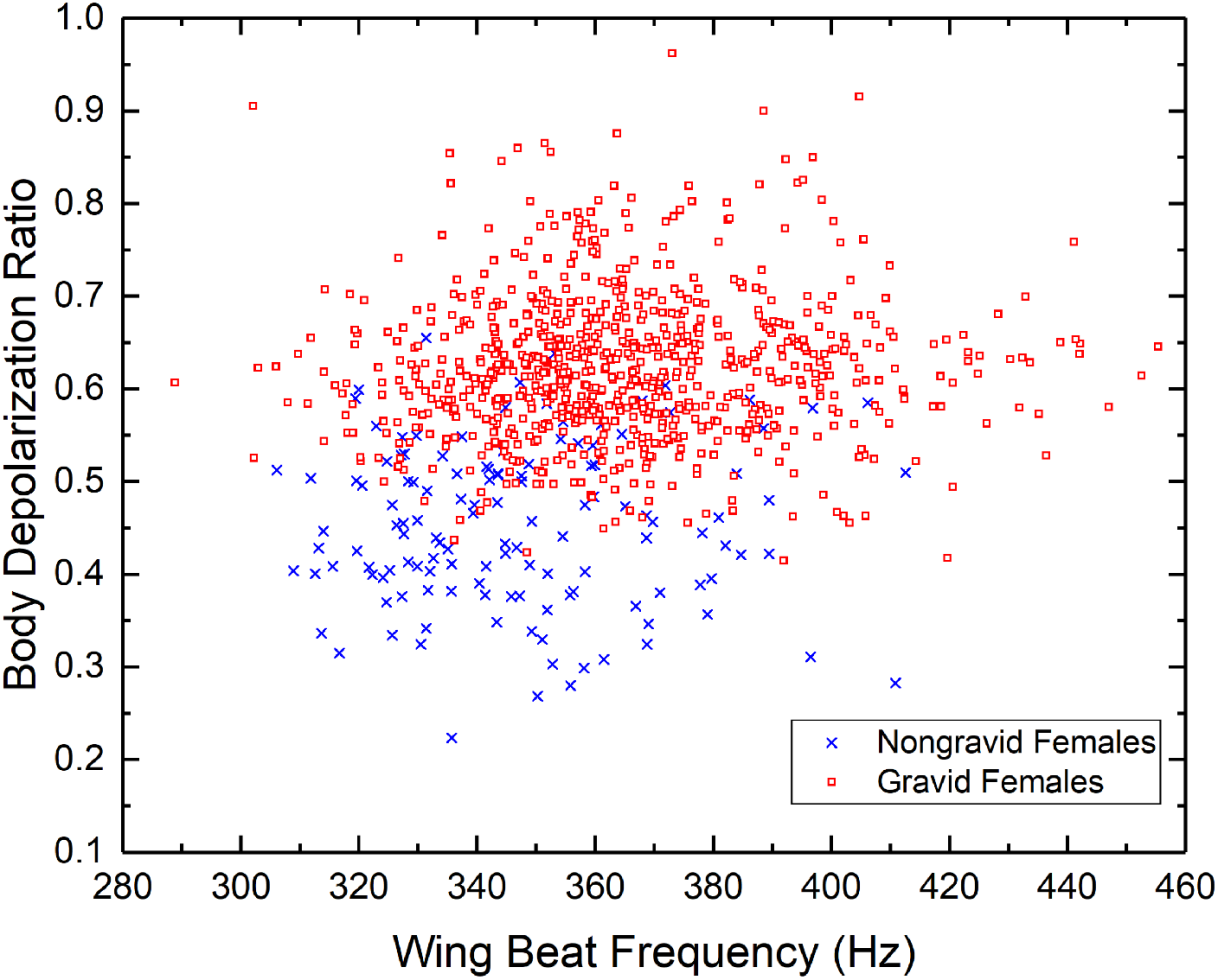

## Graphical abstract

Improving the survey of mosquito populations is of the utmost importance to further enhance mitigation techniques that protect human populations from mosquito‐borne diseases. This work demonstrates that gravid mosquitoes (carrying eggs) can be identified from changes in their spectral and polarimetric backscatter cross sections. Entomological lidars and/or optical sensors can take advantage of these optical properties to improve their ability to remotely monitor insect ecosystems.

## 1 INTRODUCTION

Reducing blood sucking mosquito populations is one of the key methods for preventing the spread of mosquito‐borne diseases such as malaria, yellow fever, dengue, West Nile and Zika virus to name a few. According to the Centers for Diseases Control and Prevention (CDC, USA), “improved surveillance system”, “monitoring and mitigation of threats” along with the use of “targeted strategies” are needed in order to prevent the spread of mosquito borne disease such as malaria 1. Still according to the CDC, this disease alone is responsible for 445 000 deaths in 2016. It is therefore paramount to further improve fine‐scale detection of mosquito populations, and in particular, breeding habitat or high‐activity areas for targeted mitigation techniques.

New and innovative optical methods have known recent improvements in the field of entomology 2, aiming at better understanding insect population dynamics without the need to capture or disturb the natural behavior of the studied specimens. Physical traps allow for a very extensive study of the captured specimens with almost 100% identification accuracy and have been successfully used in a great number of studies. However, entomological lidars and optical systems have great potential for the study of insect populations remotely and in real‐time. They can monitor thousands of insects in the span of a few hours 3 without the need for expensive and laborious taxonomic analysis in a laboratory. Furthermore, such systems can record the flight trajectory or position of the insects in real time 4, 5, 6, 7, 8, 9, 10, enabling the identification of high‐risk areas for targeted mitigation techniques, and the study of predator‐prey dynamics or mating behavior if coupled with species and sex identification. Such identifications are possible due to predictor variables retrieved from interactions of the insect with light. For instance, the wing beat frequency and harmonics content of flying insects retrieved from entomological lidar signals have been used to differentiate between species 5, 6, 9, 10, 11, 12, 13, 14, 15, 16, 17. The wing beat frequency has also been used to discriminate sex and identify mosquito species in particular 7, 16, 17, 18, 19, 20, 21, 22. In addition, insects from different families or species present different optical properties. Insect bodies may present various degrees of melanization and water content, while wings have different pigments, impacting the diffuse reflectance, and thin film layered structures, making wings iridescent 5, 6, 10, 11, 12, 23. The spectral and polarimetric features derived from a mosquito's interaction with light have been studied 7, 17, 24 in an effort to improve the specificity of entomological lidar. For mosquitoes in particular, the differentiation between species and sex is of the utmost importance since some species are known disease vectors to human populations, such as *Aedes albopictus* (Skuse) for the dengue fever or *Culex pippins* and *Aedes vexans* (Meigen) for the West Nile virus, while other species rarely consider humans as possible hosts 25. Furthermore, the identification of sex differentiates females from males which is crucial for sterile insect mitigation techniques 26 but also because only the female displays a biting behavior for reproductive purposes. Over the course of their life, females present an entirely different behavior than males, mainly while they search for a blood meal, digest it, during the embryogenesis and when looking for oviposition sites 27, 28, 29. Also, as males tend to emerge earlier than females, sex ratios can be used as a relative measure of adult mosquito emergence. Evaluating insect populations and understanding their circadian rhythms is critical to reduce human exposure to peak activity of mosquitoes, as it can be used to encourage caution or implement mitigation techniques in a specific area at specific time.

In this contribution, we investigate the differentiation of gravid female (carrying eggs) from non‐gravid female (not carrying eggs) mosquitoes. This information can be used to identify females potentially carrying infectious diseases, possible breeding habitat, predict an increase in the mosquito population and provide a better overall understanding of the ecosystem dynamics. Detecting freshly blood‐fed females could provide similar information on the ecosystem, however, females tend to rest while digesting, and it is therefore very unlikely to have them transiting through the laser beam. With the exception of a very few autogenous species, in order to become gravid, a female mosquito must have had a blood meal in the previous days as the complete digestion of the blood meal and the development of the ovaries takes between 69 and 97 hours 30. Adult females lay their eggs in various ways depending on the species. For most species, females will lay around 100 to 300 eggs, in stagnant water. The eggs will then hatch as soon as the conditions, water and temperature are favorable. Therefore, monitoring the gravid female population together with atmospheric conditions may provide information on when newly emerged mosquitoes are likely to become active, which can be used to anticipate the risk of exposure to infectious diseases.

This experiment is done in laboratory conditions with a prototype operating at short range (≈4 m). As such, the current prototype is not built for field experiments but solely for laboratory measurements in a control environment. The system is built to mimic a larger portable system, adapted for field measurement, which would be capable of reaching greater range (≈100 m) utilizing a telescope with larger collecting optics than the current prototype. The optical layout of the emitting and receiving parts would be identical to this prototype. The size for each part is less than 25 × 25 × 25 cm^3^ with a weight below 2 kg, which makes the system suitable to be mounted on the portable telescope. An estimate of the minimum cost for a portable long‐range system would vary between 5000 and 10 000 USD, mostly depending on the quality of the laser sources and telescope primary mirror. The identification, also referred to as classification, is conducted using predictor variables such as the wing beat frequency, depolarization ratios and optical cross section ratios, extracted from the measurements of the dual‐wavelength polarization‐sensitive optical system. The classifier itself is based on supervised machine learning and relies on a linear discriminant analysis (LDA) technique 31, 32.

## 2 METHODOLOGY

### 2.1 Experimental methodology

**Figure 1:**
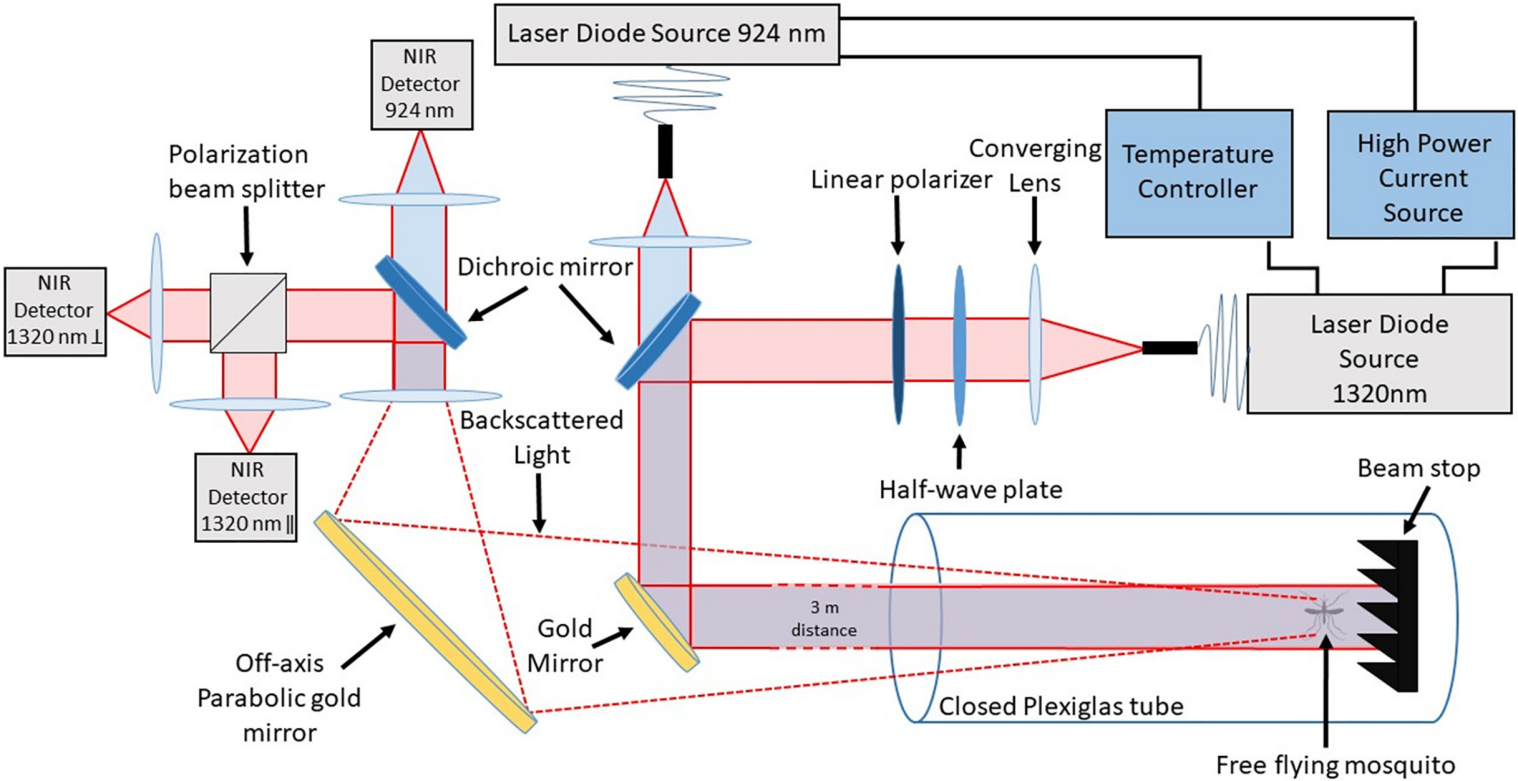
Optical layout of the dual‐wavelength polarization‐sensitive infrared optical system

Figure 1 illustrates the layout of the optical system. This system, described in more detail in a previous article 11, simultaneously records three different channels from two continuous wave (CW) laser diodes in the short‐wave infrared (SWIR) and near‐infrared (NIR). The scattering efficiency in the SWIR spectral range is rather unaffected by water and melanin, which is the most common chromophore in insect wings and body, while NIR scattering will vary with the degree of melanization. Thus, the two wavelengths were chosen in the NIR and SWIR spectral ranges in order to maximize the contrast between their respective optical cross sections. The first channel records the light wave backscattered by insects from a 924‐nm CW laser diode (L4‐9891510‐100M; Lumentum, Milpitas, California), denoted I924. The second and third channels collect the backscattered light from a 1320‐nm CW laser diode linearly polarized (4PN‐116; SemiNex, Peabody, Massachusetts). A polarizing beam‐splitter cube separates parallel (//) and cross‐polarized (┴) backscattered signals with respect to the laser polarization plane, resulting in channels I_1320,//_ and I_1320,┴_. As such, the system is rather similar to dual‐wavelength polarization sensitive lidars commonly used to monitor and study atmospheric aerosols 33, 34. For both laser diodes, laser beams are superimposed, using a dichroic mirror, to follow the same optical path and expended to reach 2.54 cm full‐width half‐maximum diameter to increase the likeliness of a mosquito transiting through the beams while decreasing the beam divergence. The backscattered intensity on all three channels is measured by Indium Gallium Arsenide (InGaAs PDA20CS; Thorlabs, Newton, New Jersey) amplified photodetectors with a 67 kHz bandwidth and recorded with a 16 bit 250 MS/s 125 MHz bandwidth digitizer (M4i4420‐x8; Spectrum, Stamford, Connecticut). Data were pre‐averaged and acquired at a sampling rate of 30 517 Hz by the acquisition software.

All signals of mosquitoes transiting through the laser beams, hereafter referred to as “events”, are measured in a laboratory‐controlled environment where the actual species, sex and gravidity of the specimen is known. Several mosquitoes of the same species, sex and gravidity are introduced into the enclosure chamber simultaneously. They are then free to fly within the closed plexiglass tube and to cross the laser beams.

In this study, three mosquito species of both sex were studied:

- *A albopictus* (number of males: 23, number of females: 28, total: 51), commonly called the Asian tiger mosquito, is present in Western countries such as the United States and originates from Southeast Asia and its tropical and subtropical areas. This species can be a vector of serious diseases such as yellow fever and dengue to name a few and is therefore of particular interest in respect to health agencies.
- *A vexans* (number of males: 18, number of females: 20, total: 38), widely present in Europe and with a foothold in most continents, including North America. These mosquitoes can spread the deadly West Nile, Rift valley fever or St. Louis encephalitis viruses. They also display an aggressive biting behavior towards humans and are a potential contributor to the recent North America Zika outbreak.
- *Culex Genus* (mixed species) (number of males: 36, number of females: 30, number of gravid females: 63, total: 129), this Genus includes several species that are vectors of disease. *Culex* is spread all across the globe and is the most predominant Genus in the North America mosquito population. Our samples primarily contained mixed groups of morphologically similar *C pipiens* Linnaeus and *Culex restuans* Theobald.

Mosquitoes came from the Hudson Regional Health Commission, Mosquito and Vector Control. They were field collected as larvae from various locations around Hudson County, NJ, and reared in plastic trays (ca. 200 larvae/tray) in 1 L of deionized water with 0.3 g of brewer's yeast provided on alternate days. After eclosion, adults were housed in 30 x 30 x 30 cm^3^ aluminum screen cages at 26°C, 75% relative humidity, with a 16:8 hour L:D photoperiod and provided a 10% sucrose solution. During measurements, the mosquitoes were at a temperature of 22 ± 1°C and between 30% and 80% relative humidity. All species were studied from after they hatched until they died generally 12 to 18 days later. Gravid Culex mosquitoes were collected from Secaucus, NJ, with a Center's for Disease Control gravid trap 35 (John W. Hock Co., Gainesville, Florida) baited with a hay infusion 36. Collected *Culex* mosquitoes were visually identified only to genus to avoid damage to the specimens but were most likely *C pipiens* Linnaeus and *C restuans* Theobald. Gravidity was visually confirmed by viewing egg masses through the cuticle of the mosquitoes.

### 2.2 Predictor variable and machine learning classification

The optical layout allows to record the backscattered signals on the three channels for which the contributions of the wings (index w) and body (index b) can be differentiated 6, 17, 37, resulting in six measured backscattered signals each proportional to the optical cross section *σ*_924,*w*_, *σ*_924,*b*_, *σ*_1320,//,*b*_, *σ*_1320,//,*w*_, *σ*_1320,⊥,*b*_ or *σ*_1320,⊥,*w*_. The backscattered signals are calibrated using Lambertian white targets of known size to retrieve the optical cross section in mm^2^. Signal intensities also vary with the position of the insect with respect to the Gaussian spatial profile of the beam, namely the position coefficient ρ which is equal to the ratio between the power density at the insect position and the maximum power density in the beam. The backscattered intensity will be maximum when the insect is at the center of the Gaussian beam (i.e. maximum power density, *ρ* = 1) and minimum when the insect is outside the beam (i.e. *ρ* = 0). For every event, the wing beat frequency and its harmonics can be retrieved through a fast Fourier transform or the Welch method on the wing backscattered signals. The wing beat frequency has been shown to differentiate males from females and to a lesser extent differentiate species of mosquito 7, 16, 17, 18, 19, 20, 21, 22. Linear depolarization ratios δ are retrieved from the parallel and cross‐polarized backscattered signals, as defined by Equation 1.

(1) $$\delta =G∙\frac{I_{1320,⊥}}{I_{1320,//}}=\frac{\sigma_{1320,⊥}}{\sigma_{1320,//}}$$

Where G is our calibration constant between our parallel and perpendicular channel obtained using the calibration method described in Alvarez et al 38.

In addition to the wing beat frequency, other predictor variables can be extracted from the backscattered signals. Once calibrated, the ratio between wings or body signals from the three channels is equivalent to the ratios of their optical cross sections. The predicator variables used in this classification are based on every possible combination of the ratio between the six optical cross sections. As one can expect, some are more efficient that others, in the case of the discrimination of gravid females, the most efficient predictor variables are the body depolarization ratio, and the wing beat frequency. On the other hand, predictor variables related to wings, such as wing depolarization ratios or wing spectral ratios, display little to no variation between gravid and non‐gravid females, making them inefficient predictor variables. Predictor variables are described in detail in previous work 11.

The value distribution of the predictor variables is used to construct a classifier that uses the in‐between class differences to differentiate predefined classes (understood as species and/or sex and/or gravidity). In this contribution, the classifier is built using supervised machine learning based on LDA method. Un‐supervised machine learning may be as well suitable for such application; however the number of recorded signals in this experiment, 1375, is currently too low to properly train an un‐supervised model. The LDA method was chosen for different reasons: first, for its relative simplicity of implementation, quick training time and natural support of more than two simultaneous classes. Second, LDA involves a dimensional reduction and reducing the number of predictor variables can improve the prediction accuracy 11. This dimension reduction process involves both the optimization of the separation between classes and the minimization of the within‐class variance. This reduction is therefore effectively screening out the predictor variables that are less suitable, or even possibly unsuitable, for the specific classification task without requiring any prior knowledge about the predictor variables meaning or relative importance.

In order to characterize the efficiency of the classifier, a leave one out cross‐validation is applied 39. To this effect, the data are divided in training and testing sets. Events from each class are randomly separated into different subset of equivalent sizes. All but one subset are used for training and the remaining subset is used for testing. The process is repeated until every subset has been used as a testing set exactly once.

Supervised machine learning classifiers are most efficient when the training events are numerous. Yet, it is important to ensure a relative balance between the number of events in each class as imbalanced classes can lead to a decrease in a classifier performance 40. Since different classes can have a different total number of events, a random under‐sampling method was used 41, 42. Briefly, it consists of randomly eliminating events from classes with most events until the desired class distribution is achieved, which here, is the same number of events in all classes. The downside of this method is the potential loss of information from the removed events. Yet random under‐sampling, despite its relative simplicity, has been shown to be an effective resampling method and can be equally effective as more elaborate resampling techniques 40, 42.

After applying the random under‐sampling and leave one out cross‐validation, the results of the prediction are evaluated. As the experiment is conducted in a laboratory‐controlled environment the correct class of every event is known a priori and can be compared to the predicted class by the LDA classifier. In this contribution, four different metrics were chosen to evaluate the classifier. The overall accuracy of the classification which is the percentage of correctly predicted events throughout all classes (Equation 2), the recall which is the percentage of events from a given class to be predicted as such (Equation 3), the precision which is the percentage of events predicted as a given class that actually are of this class (Equation 4 and the F1 score (Equation 5) that is a common evaluation metric for multi‐class classifiers.

(2) $$\mathrm{OAC}=\frac{\sum_{i}N_{\mathrm{ii}}}{\sum_{i}N_{\mathrm{ii}}+\sum_{i}N_{i\neq j}},$$

(3) $$\text{Recall}\left(i\right)=\frac{N_{\mathrm{ii}}}{\sum_{j}N_{\mathrm{ij}}},$$

(4) $$\text{Precision}\left(i\right)=\frac{N_{\mathrm{ii}}}{\sum_{j}N_{\mathrm{ji}}},$$

(5) $$F1\;\text{score}\left(i\right)=2∙\frac{\text{Recall}\left(i\right)∙\text{Precision}\left(i\right)}{\text{Recall}\left(i\right)+\text{Precission}\left(i\right)},$$

where *N*_*ii*_ is the number of all the correct predictions for the class i, *N*_*ij*_ the events of the class i predicted as class j, *N*_*ji*_ the events of the class j predicted as class i and *N*_*i* ≠ *j*_ the number of all the wrong predictions for the class i (either a class i predicted as j or a class j predicted as i).

Those metrics can be applied and calculated for any given LDA classifier. Yet, because of the random under‐sampling method and the limited number of accessible training data sets, the randomly selected pools of events can lead to different results. This variability is limited to a few percent difference between the best and worst results. To this effect, 1000 LDA classifiers were created using random under‐sampling and evaluated using leave one out cross‐validation. The final characterization of each metric, presented in section 3.2, is the average value calculated from the leave one out cross‐validation results of the 1000 LDA classifier. Repeating the evaluation process induces a better characterization of the classifier and allows for an evaluation of the variability of the average on each metric with a 95% confidence interval using standard error extracted from the standard deviation.

## 3 RESULTS AND DISCUSSION

### 3.1 Data analysis

**Figure 2:**
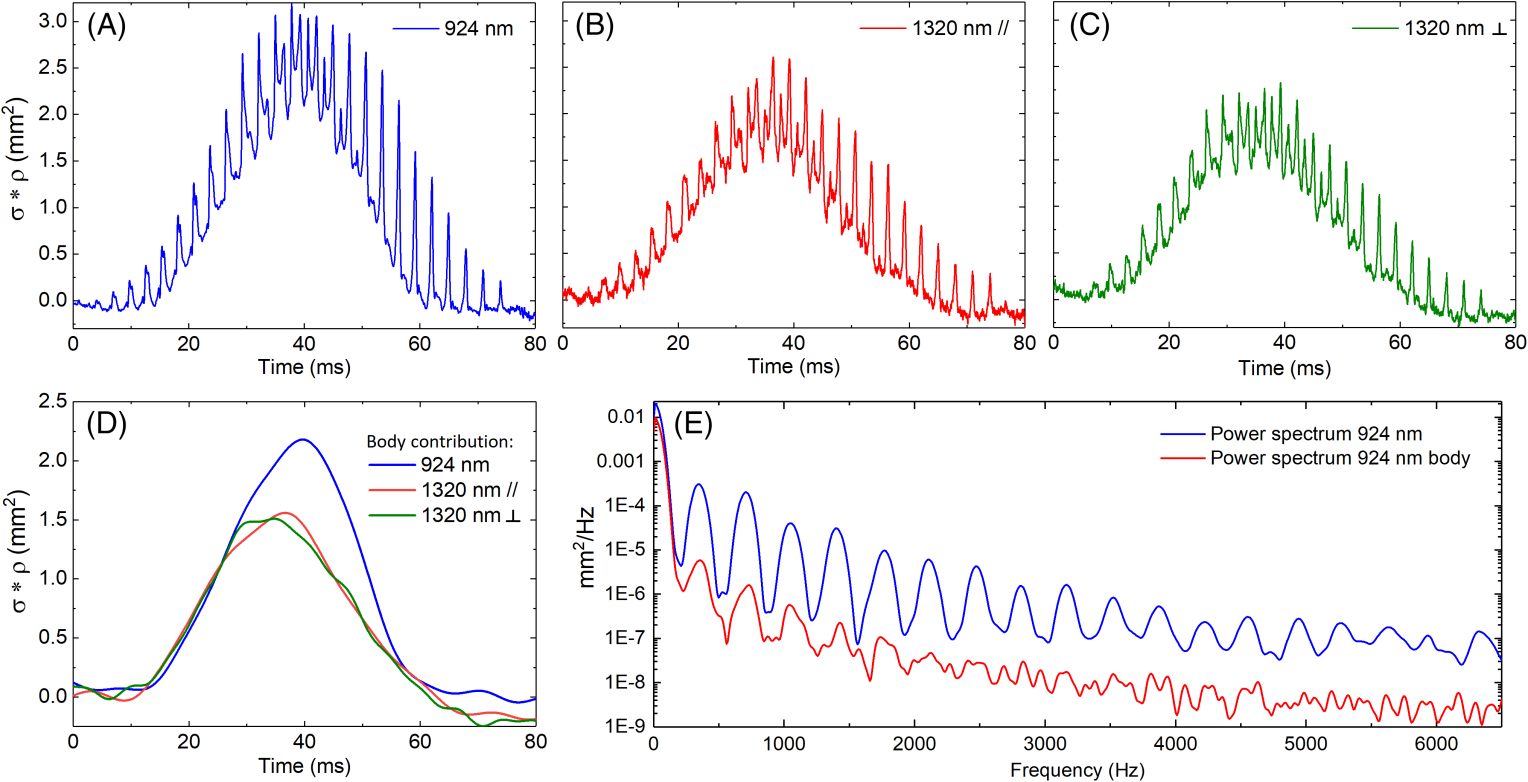
Signals of one mosquito transiting through the laser beams as recorded by the three channels. A, B and C present the weighted optical cross section (*ρ, expressed in mm^2^) for, respectively, the 924, 1320 parallel and 1320 nm perpendicular channels. D presents the body contribution for each signal. E presents the power spectrum of the signal and body signal at 924 nm, showing the wing beat frequency at 348 Hz and its harmonics

In this section, an example of a recorded event by the three detection channels is presented in Figure 2. From the recording on all three channels, the predictor variables are extracted following the methodology described in Section 2.2. This is applied to all of the 1375 recorded events and then used to train and test the LDA classifier for different class separation. First, the aim is to separate gravid females of the *Culex* genus from non‐gravid females of the same genus, demonstrating the ability of the system to identify the gravidity of a specimen. Second, we extend the classification to include both male and female of the *Ae. albopictus* and *Ae. vexans* species and the *Culex* genus in order to show that even among a wider pool of species the gravidity of the female *Culex* can still be retrieved efficiently.

In Figure 2, one example of a mosquito event is presented. All three channels are simultaneously recorded, and the predictor variables extracted from their analysis. The more intuitive feature, presented in Figure 2A‐C, is the periodicity in the signal that is due to the wing orientation rapidly changing during the mosquito transit. This feature can be observed by the periodic succession of sharp intensity peaks. This allows for the retrieval of the wing beat frequency and harmonics (Figure 2E) and the discrimination between the wing and body contributions. Figure 2D presents the body contributions, the ratio between the 924 and 1320 nm body cross section slightly changes over the course of the transit, potentially caused by the insect orientation changing over time and, therefore, showing parts of its body with different degrees of melanization. Both body and wings optical cross sections appear to be higher in the SWIR spectral range than in the NIR. The maximum optical cross section of wings is on average 1.6 higher while the body cross section is 2.5 higher.

**Figure 3:**
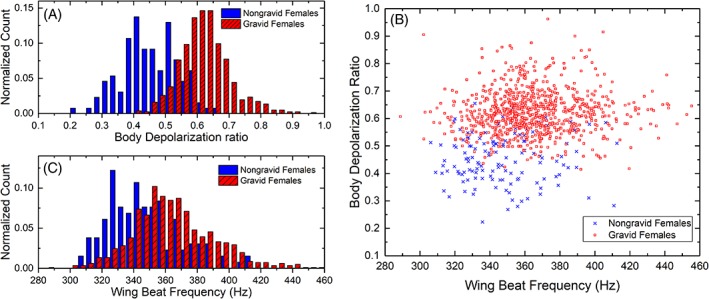
Normalized histogram for the depolarization ratio of the body (A) and the wing beat frequency (B) for the gravid and non‐gravid female of the Culex Genus. Scatter plot for both aforementioned predictor variables and the same classes (C)

As previously studied 7, 17, 24, dual‐wavelength and polarization‐sensitive measurements can be useful, in addition to the wing beat frequency, for the species and sex identification of mosquitoes. The work presented here also demonstrates that they can contribute to the identification of the gravidity of female mosquito from the *Culex* Genus. As displayed in Figure 3, the depolarization ratio measured from the body of gravid females is higher on average than for non‐gravid females, respectively, 0.62 and 0.45 which represent an increase of 38%. In addition, an increase of 5.2% can also be observed in the average wing beat frequency, from 347 Hz for non‐gravid females to 365 Hz for gravid females due to the insect compensating for the increase of its weight 43, 44. Using the formulae provided by M. Deakin 45 relating the wing beat frequency and the mass m of the insect, the wing beat frequency increases with m^0.3^ leading to an increase of mass between gravid and non‐gravid of approximately 0.91 mg. A similar estimation of the change in mass, using the depolarization ratio to infer the increase in optical path‐length in the scattering medium 46 and therefore the increase in volume and mass, leads to an increase in mass of 0.47 mg. The Culex female lays between 100 and 300 eggs, each fully grown egg weighting between 10 and 15 μg 47. The retrieved increase in mass seems consistent considering that measurements were made on gravid females at various stages of the embryo development for which a lower egg mass is expected. Furthermore, the number of eggs is correlated with the volume of the blood meal, 48; therefore, a smaller blood meal could result in a reduced among of eggs. This difference on both predictor variables is then used, along with other predictor variables, to train the LDA classifier allowing the differentiation of gravid from non‐gravid mosquitoes, as described in Section 2.2.

### 3.2 Female gravid identification

The first part of the classification scheme is directed towards the differentiation between gravid and non‐gravid *Culex* mosquitoes. To characterize the effectiveness of the LDA classifier for this two‐class system, the calculated evaluation metrics are recall, precision, F1 score and overall accuracy, as described in Section 2.2, and are displayed in Table 1.

**Table 1:** Results of the two class LDA classification

	Average precision	Average recall	Average F1 score
*Culex* female non‐gravid	87.1 ± 0. 16%	85.9 ± 0.14%	86.5 ± 0.13%
*Culex* female gravid	86.1 ± 0.13%	87.3 ± 0.17%	86.7 ± 0.13%

*Note*: Recall is the percentage of events from a given class to be predicted as such (Equation 3). Precision is the percentage of event predicted as a given class that are actually of this class (Equation 4). F1 score evaluates the efficiency of multi‐class classifiers (Equation 5). All results are given within the 95% confidence interval.

Abbreviation: LDA, linear discriminant analysis.

These results show that predictor variables extracted from dual‐wavelength polarization‐sensitive measurements can be reliably used to train an LDA classifier for the purpose of differentiating gravid mosquito from non‐gravid mosquito. When only the wing beat frequency is considered the recall and precision are lower, by 26.9% and 21.3%, respectively, meaning that the polarization‐sensitive measurements greatly improve the identification of gravidity. In addition to the class‐specific metrics presented in Table 1, the overall classification accuracy can be calculated (Eq. 2). For this classification, the average overall accuracy is 86.6% ± 0.13% which is 22.1% better than the classification that only includes the wing beat frequency as a predictor variable.

The second scheme is also directed towards identifying gravid Culex mosquitoes among a broader pool of possible classes (Table 2), as expected in field measurements. This is done by including male and female (all non‐gravid) *Ae. albopictus*, *Ae. vexans* and *Culex* mosquitoes to the previously described two class classification.

**Table 2:** Results of the 7‐class LDA classification

	Average precision	Average recall	Average F1 score
Albopictus male	84.6 ± 0.22%	75.2 ± 0.30%	79.6 ± 0.22%
Albopictus female	77.1 ± 0.37%	70.0 ± 0.38%	73.2 ± 0.31%
*Culex* male	76.3 ± 0.37%	64.6 ± 0.29%	69.8 ± 0.25%
*Culex* female	79.3 ± 0.31%	79.4 ± 0.36%	79.2 ± 0.26%
Vexans male	72.4 ± 0.24%	77.2 ± 0.25%	74.7 ± 0.20%
Vexans female	65.9 ± 0.30%	79.6 ± 0.35%	72.0 ± 0.26%
*Culex* female gravid	78.9 ± 0.31%	83.7 ± 0.33%	81.1 ± 0.25%

*Note*: All classes are to be considered non‐gravid unless otherwise specified. Results are given within the 95% confidence interval.

Abbreviation: LDA, linear discriminant analysis.

These results demonstrate that even when the gravid females are identified within a larger pool of possible classes, namely other mosquito species and sex, they can still be separated accurately. Moreover, the classifier still performs efficiently for classes other than the gravid one with an overall accuracy of 75.7 ± 0.13%.

## 4 CONCLUSION

In this contribution, a dual‐wavelength polarization‐sensitive optical system is used in laboratory conditions to identify the species, sex and gravidity of the free flying mosquitoes in its field of view at a distance between 3 and 4.25 m. From the backscattered signals, 18 predictive variables, such as wing beat frequency, depolarization ratios and backscattering coefficient ratios, are extracted and used to train a classifier using supervised machine learning based on LDA. The trained classifier is then able to identify the species and sex of the free flying specimen and also if a female is carrying eggs.

When the specimen is known to be a female of the *Culex* genus the classifier is able to infer the gravidity with a precision of 86.1% meaning that 86.1% of the females predicted as carrying eggs actually were, and a recall of 87.3% meaning that 87.3% of all the female carrying eggs were identified as such.

In the second part, a broader approach, closer to what can be expected in an actual field measurement situation, was tested. In this case, different species and sex are included in the classification to evaluate if the gravidity of the female could still be retrieved in a more complex situation. The measurements were performed in a laboratory‐controlled environment where both sexes of the A*e. albopictus* and *Ae. vexans* species and of the *Culex* genus were introduced in the classification to mimic a natural on‐field situation where more than one species and sex of mosquitoes can be expected to be present at the same time. With this case, the recall and precision for the gravid females of the *Culex* genus reaches 83.7 and 78.9% respectively.

The main focus of this contribution is to identify the gravidity of female mosquitoes, yet identifying the species and sex is still important for entomological sensors. The species and sex identification were also performed, in addition to the gravidity, and results show the overall identification accuracy to be 75.7% demonstrating that retrieving the gravidity along with species and sex is a possibility. Therefore, both species/sex and gravidity identification can be conducted simultaneously improving the value of entomological lidar and optical system even further.

While a higher accuracy can be achieved using physical traps, entomological lidars and optical systems can process a much higher number of insects, remotely and in real‐time. As such, we believe that optical systems may be a viable alternative or complementary methodology to study insect ecosystems and/or monitor the population and behavior of mosquito species related to infectious diseases.
